# Maternal Obesity: An Obstetric Risk

**DOI:** 10.7759/cureus.29345

**Published:** 2022-09-19

**Authors:** Amala Sunder, Bessy Varghese, Basma Darwish, Noor M Shaikho, Abeer AlSada, Haya Albuainain, Salem Alrowaijeh, Shooq Abdulla Al-Khalifa, Ameena Khalid Bughamar, Nawal Dayoub

**Affiliations:** 1 Obstetrics and Gynaecology, Bahrain Defence Force Hospital, West Riffa, BHR; 2 Anaesthesia, Bahrain Defence Force Hospital, West Riffa, BHR; 3 Obstetrics and Gynaecology, Assisted Reproduction in Gynecology Center, London, GBR

**Keywords:** induction of labour, antepartum hemorrhage, intra uterine growth restriction, gestational hypertension, gestational diabetes mellitus, body mass index

## Abstract

Objective: To analyze the obstetric risks and to evaluate the effects of maternal obesity during pregnancy and postpartum period.

Method: This is a retrospective study of pregnant women with a BMI of more the 30 conducted at Bahrain Defence Force Hospital, West Riffa, Bahrain, from September 2019 to August 2020. Data includes demographic characteristics, and course of pregnancy from gestational age 24 weeks, through intrapartum to the postpartum period. Adverse maternal effects and delivery complications were the primary study outcomes. The BMI was calculated at the time of the booking visit. Comparative analysis was done to calculate the odds of each outcome taking a non-obese group (BMI less than 30) as a reference.

Results: The total number of pregnant women studied was 2972, out of which 1657 had BMI ≥30. In our study, women with high BMI were older (p<0.0001). High BMI was associated with high parity and higher miscarriage history. High BMI increased the risk of developing hypertension (OR 2.5; 95%CI 1.1-5.3). This analysis also found that high BMI was associated with increased risk of antepartum hemorrhage (OR 2.4; 95%CI 1-5.4), postpartum complications (OR1.6; 95%CI 1.1-2.2), and a hospital stay of more than five days (OR 1.6; 95%CI 1.3-2). High BMI patients were less likely to have Intrauterine growth restriction (OR 0.6; 95%CI 0.3-0.9). High BMI patients did not have an increased risk of gestational diabetes mellitus, induction of labor, or caesarean birth.

Conclusion: Higher BMI pregnant women are associated with higher incidences of hypertension. The high BMI group also had a significant relationship with antepartum hemorrhage and postpartum length of stay.

## Introduction

The global increase in obesity and the accompanied morbidities lead the way to study the consequences of obesity in pregnancy [1]. A Middle East systemic review by Okati et al. revealed the elevated prevalence of overweight and obesity as well as the rising obesity with age [2]. The Kingdom of Bahrain's prevalence of obesity is rising at a standard annual rate of 1.13%. It was 29.7% in 1997 and rose to 36.8% by 2016 [3]. Obesity is more frequently observed among women of reproductive age, and it is associated with obstetric risks [4,5]

Generally, obesity is calculated at the booking visit during pregnancy and categorized as obese, when BMI is more than 30 [6]. Maternal obesity is associated with severe maternal and perinatal mortalities and morbidities, which include gestational diabetes mellitus (GDM), gestational hypertension, preeclampsia, and stroke [7-9]. Intrapartum complications are prolonged labor, shoulder dystocia, instrumental deliveries, increased cesarean delivery rate, and anesthesia complications. Postpartum complications include postpartum hemorrhage, puerperal pyrexia, wound infection, venous thromboembolism, longer hospital stay, and postpartum depression. Obesity-related fetal risks are miscarriages, birth defects, macrosomia, unexplained stillbirths, neonatal intensive care unit (NICU) admission, and neonatal deaths [10,11]

It can be challenging to care for obese women through pregnancy such as in the screening of anomalies, antenatal care, fetal surveillance, monitoring during labor, delivery, and anesthesia. A unit with an organized obstetric team, equipment, facilities, risk assessment, and management policies is required to care for these women during pregnancy [12]. In spite of the high risk, obesity in pregnancy can be managed in the form of diet, exercise, thorough monitoring of the antenatal period, and organized delivery plans.

Given the importance of assessing the effect of obesity on antenatal and postnatal outcomes, this study was conducted to assess the risks associated with increased BMI in pregnant patients from the Middle East. The objective was to identify any association between obesity and maternal outcome (antenatal, intrapartum, and postnatal). Once potential increased risk was identified, a multivariable logistic regression analysis was conducted to assess the risk and eliminate any contributing co-variables.

## Materials and methods

This is a retrospective data analysis of pregnant women who had maternity care in our unit at Bahrain Defense Force Hospital, West Riffa, Bahrain, from September 2019 till August 2020. The study was approved by the Royal Bahrain Defence Force Hospital Ethical Committee (approval BDF/R&REC/2020-421). Electronic records of women were obtained from the labor room and data protection policy rules were followed. Data were anonymized. Pregnant women with delivery at a gestational age of less than 24 weeks were excluded from the study. Any pregnant women who were infected with coronavirus disease 2019 (COVID-19) during the study period were excluded to reduce the bias in the complications. Also, all patients with incomplete records were excluded.

BMI at the booking antenatal visit in our antenatal clinic/health center/GP clinic was set for the study. They were divided into two groups, BMI ≥ 30 and BMI < 30. Variables, such as maternal age, parity, current obstetric details, past obstetric complications such as history of previous cesarean sections, miscarriages, multiple pregnancies, in vitro fertilization (IVF) pregnancy, ectopic, preeclampsia, eclampsia, GDM, obstetric cholestasis, postpartum hemorrhages, maternal comorbidities such as hypertension, gestational hypertension, eclampsia, preeclampsia, diabetes, GDM, obstetric cholestasis, bariatric surgery, and the association of BMI were studied.

We followed our hospital protocols for the identification of morbidities, which are based on the American College of Obstetricians and Gynecologists (ACOG) guideline recommendations for screening GDM and criteria of eclampsia, preeclampsia, and gestational hypertension. Preeclampsia is defined when the systolic blood pressure is 140 mm Hg or more or diastolic blood pressure is 90 mm Hg or more on two occasions at least four hours apart after 20 weeks of gestation in a woman with a previously normal blood pressure along with proteinuria, which is defined when 300 mg or more per 24-hour urine collection or protein/creatinine ratio of 0.3 mg/dl or more or dipstick reading of 1 and more, or in the absence of proteinuria, new-onset hypertension along with deranged hematological tests, impaired liver function tests/renal function tests. In the absence of proteinuria, high blood pressure is categorized as gestational hypertension. Eclampsia is any new onset of convulsions in the absence of disorders such as epilepsy and cerebral pathology. To identify GDM, we do a two-hour 75 g glucose tolerance test at 24-28 weeks of gestation for all pregnant women; if a woman has a previous history of GDM, we do the screening test at the booking visit. Detailed study of antenatal course, such as miscarriage, risk of GDM, hypertension, antepartum hemorrhage, intrauterine growth restriction (IUGR), and amniotic fluid index, the delivery details such as mode of delivery, gestational age at delivery, stages of labor, perineal tears, and shoulder dystocia, and postpartum course, which includes estimated blood loss, postpartum sepsis, and length of stay in days were assessed.

Statistical analysis

Statistical analysis was done using StatsDirect (Version 3.3.5 (22nd March 2021); StatsDirect Ltd, Birkenhead, United Kingdom). The first assessment was done on the data to explore differences between groups with regards to basic characteristics such as age, parity, previous obstetric history, presence of comorbidities such as any positive medical history, smoking, IVF conception, and bariatric surgery. Furthermore, antenatal, delivery, and postnatal courses were also compared between the groups. A two-sided unpaired t-test was used for normally distributed continuous variables, a two-sided Mann-Whitney test was used for nonparametric variables, and Chi-square/Fisher's test was used for categorical variables. Then multiple logistic regression analysis was performed to identify the effect of obesity on the risk of developing GDM, hypertension, antepartum hemorrhage, and IUGR during pregnancy after adjusting for all other contributing risk factors such as age, parity, miscarriage, any comorbidity, multiple pregnancies, smoking, previous cesarian section/bariatric surgery, and IVF pregnancy. Also, multivariable logistic regression was performed to assess the effect of high BMI on the risk of having induced labor, cesarean birth, postpartum complications, and longer hospital stay, after adjusting all previously mentioned potentially-contributing risk factors. Results are presented as OR with 95%CI and p-value. A p-value < 0.05 was considered statistically significant.

## Results

Out of the total 2972 patients recruited for the study, 1657 had BMI ≥30, which represents more than half of the patients in the study (55.8%). High BMI patients were older with a mean age of 31.1 compared to normal BMI patients with a mean age of 28.4 (p<0.0001). Patients with high BMI had more than one previous delivery and more previous miscarriages compared to normal BMI patients 62.4% vs 48.5% and 27.8% vs 24.6%, respectively. High BMI patients also had a significantly higher history of twin and previous cesareans 3.6% vs 1.2% and 21.7% vs 12.8, respectively. High BMI patients included more smokers and had a higher presence of co-morbidity and IVF conception. Interestingly, more patients with previous bariatric surgery were noted in the high BMI group 1.9% vs 1.1 %. There was no difference in previous poor obstetric history, ectopic, eclampsia, and obstetric cholestasis between the groups (Table 1). 

**Table 1:** Basic characteristics H/O: history of; IVF: in vitro fertilization; NS: not significant

	BMI ≥30 (n=1657)	BMI < 30 (n=1315)	p-value
Maternal age	31.1±5.7	28.4±5.6	<0.0001
Primigravida	267(16.1)	321(24.4)	<0.0001
Parity 1	356(21.5)	356(27.1)	0.0004
Parity >1	1034(21.5)	638(48.5)	<0.0001
Miscarriage	460 (27.8)	323 (24.6)	0.04
Past obstetric history	58 (3.5)	46 (3.5)	NS
H/O twins	60 (3.6)	16 (1.2)	<0.0001
H/O ectopic	8 (0.5)	1 (0.08)	NS
H/O postpartum hemorrhage	17 (1)	15 (1)	NS
H/O eclampsia	6 (0.4)	4 (0.3)	NS
H/O preeclampsia	23 (1.4)	11 (0.8)	NS
H/O obstetric cholestasis	10 (0.6)	2 (0.2)	NS
Any comorbidity	343 (20.7)	216 (16.4)	0.003
Smoking	12 (0.7)	1 (0.1)	0.009
Previous cesarian section	360 (21.7)	168 (12.8)	<0.0001
Bariatric surgery	33 (1.9)	14 (1.1)	0.04
IVF conception	61 (3.7)	27 (2.1)	0.009

In general, patients with higher BMI had more antenatal complications, 20.8% compared to 16.1% in the normal BMI group (p=0.001). There were more cases of GDM and hypertension in the high BMI group, 10.4% vs 7.6% and 1.8% vs 0.7%, respectively.

The analysis revealed a higher incidence of threatened antepartum hemorrhage in the high BMI group, 1.4% vs 0.6%. Remarkably, there was no difference in the incidence of pre-existing diabetes mellitus type1 and type 2 and gestational hypertension/preeclampsia toxemia (PET). The high BMI group showed a similar incidence of obstetric cholestasis, placenta/amniotic fluids abnormalities, and multiple pregnancies (Table 2).

**Table 2:** Antenatal course DM: diabetes mellitus; GDM: gestational diabetes mellitus, HTN: hypertension; GHTN: gestational hypertension; APH: antepartum hemorrhage; PET: preeclampsia toxemia; NS: not significant

	BMI ≥ 30 (n=1657)	BMI < 30 (n=1315)	p-value
Antenatal complications	345 (20.8)	212 (16.1)	0.001
Type 1 DM	8 (0.5)	3 (0.2)	NS
Type 2 DM	25 (1.5)	16 (1.2)	NS
GDM	173 (10.4)	100 (7.6)	0.008
HTN	29 (1.8)	9 (0.7)	0.01
GHTN/PET	47 (2.8)	27 (2.1)	NS
GHTN	22 (1.3)	9 (0.7)	NS
PET	25 (1.5)	19 (1.4)	NS
Threatened APH	23 (1.4)	8 (0.6)	0.04
Placenta praevia	6 (0.4)	3 (0.2)	NS
Abruption	8 (0.5)	6 (0.5)	NS
Obstetric cholestasis	7 (0.4)	11 (0.8)	NS
Prolapse	3 (0.2)	0 (0)	NS
Fibroid	8 (0.5)	6 (0.5)	NS
Oligohydramnios	41 (2.5)	34 (2.6)	NS
Polyhydramnios	20 (1.2)	16 (1.2)	NS
Multiple pregnancies	46 (2.8)	33 (2.5)	NS

High BMI patients had a lower cesarean delivery rate compared to normal BMI patients but the difference was not statistically significant (34.6% vs 36.7%). There was no difference in gestational age at delivery and fetal weight between the groups. Though the delivery complications rate was similar between the groups, the post-natal complications were higher in the high BMI group, which reflected in a longer hospital stay mean of 2.6 compared to a mean of 2.2 for the normal BMI group. On a positive note, there was no difference in re-admission rate between the groups (Table 3). 

**Table 3:** Labor and fetal outcome NS: not significant

	BMI ≥ 30 (n=1657)	BMI < 30 (n=1315)	p-value
Cesarean birth	573 (34.6)	482 (36.7)	NS
Delivery gestation age	38.3±2.1	38.3±2.1	NS
Fetal weight	3.2±0.6	3.1±0.6	NS
Delivery complications	267 (16.1)	206 (15.7)	NS
Post-partum complications	109 (6.6)	57 (4.3)	0.008
Length of hospital stay	2.6± 1.8	2.2± 1.6	<0.0001
Readmission	8 (0.5)	7 (0.5)	NS

Multivariable logistic regression was performed to assess the effect of high BMI on the risk of developing GDM, hypertension, antepartum hemorrhage, and IUGR during pregnancy. After adjusting all other contributing risk factors such as age, parity, miscarriage, any maternal comorbidity, multiple pregnancies, smoking, previous cesarian section/bariatric surgery, and IVF pregnancy, there was no association between high BMI and risk of developing any antenatal complications in general (OR 1.3; 95%CI 0.99-1.6). However, once analysis was done on specific complications such as GDM/hypertension and antepartum hemorrhage, the risk of developing hypertension was the highest in the high BMI group (OR 2.5; 95%CI 1.1-5.3) (p = 0.02). Furthermore, high BMI increased the risk of antepartum hemorrhage (OR 2.4; 95%CI 1-5.4) (p = 0.04). High BMI did not increase the risk of GDM and reduced the risk of IUGR (OR 0.6; 95%CI 0.3-0.97) (p= 0.04).

Likewise, multivariable logistic regression was performed to assess the effect of high BMI on the risk of having induced labor, cesarean birth, postpartum complications, and longer hospital stay. After adjusting all previously mentioned potentially contributing risk factors, the high BMI increased the risk of postpartum complications (OR 1.6; 95%CI 1.1-2.2) (p = 0.01). Furthermore, high BMI increased the risk of long hospital stay of more than five days (OR 1.6; 95% CI 1.3-2) (P<0.0001. High BMI did not increase the risk of induced labor or cesarean section (Table 4).

**Table 4:** Risks associated with high BM (I≥30) GDM: gestational diabetes mellitus; HTN: hypertension; APH: antepartum hemorrhage; IUGR: intra-uterine growth restriction; MOD: mode of delivery; LOS: length of stay

	OR	coefficient	95% CI	p-value
Antenatal complications	1.3	0.22	0.99-1.6	0.05
GDM	1.1	0.11	0.8-1.6	NS
HTN	2.5	0.9	1.1-5.3	0.02
APH	2.4	0.9	1-5.4	0.04
IUGR	0.6	-0.6	0.3-0.97	0.04
Artificial start of labor	1.1	0.12	0.97-1.3	NS
Postpartum complications	1.6	0.4	1.1-2.2	0.01
MOD/cesarean	0.9	-0.07	0.8-1.1	NS
LOS >5 days	1.6	0.5	1.3-2	<0.0001

## Discussion

Obesity is a growing challenge for obstetricians as risks increase with pregnancy. A conclusion of three cohort studies by Bellver et al, Wang et al., and Fedorcsak et al. identified obesity as an independent risk factor for spontaneous miscarriage in those who undergo fertility treatment [13-15]. A threefold increase in miscarriage was found with ovulation induction with gonadotropins and a fourfold increase in miscarriage with egg donation in women with a BMI of more than 30kg/m^2^ [16]. In our study, we found that women with a higher BMI had a history of more miscarriages when compared to women with optimum weight. In our study, obese women had a higher incidence of previous twins but not in the current pregnancy. Contrary to our data, a collaborative perinatal project conducted in the United States analyzed 51,783 pregnancies of which 561 were twins. The twinning occurrence was compared to maternal pre-pregnant BMI. Dizygotic twins’ incidence was 1.1% in obese women when compared to 0.5% in women with optimum weight. They concluded that without fertility drugs, maternal weight and height had a positive association with dizygotic twins in their population [17].

Insulin resistance seems to be the altered metabolic state that results in many risks in pregnancy. A study on 287,213 pregnancies relating to maternal obesity and pregnancy outcome, conducted in London by Sebire et al. concluded that the basis of many complications in maternal morbid obesity is likely to be associated with the altered metabolic state [18]. Another study on 16,102 pregnant patients found that GDM increased to 6.3% in obese women and 9.5% in morbidly obese [19]. According to our data, there was no increased risk of GDM in high BMI patients. This could be due to ethnic variation in our study population and better adherence to diet and exercise recommended at an early stage of pregnancy. A systematic review was done by Marchi and colleagues who concluded that preeclampsia, gestational hypertension, GDM, depression, instrumental delivery, cesarean section, and surgical site infection tend to occur in obese women when compared with women with healthy weight [20].

The risk of preeclampsia doubled with each 5-7kg/m^2^ increase from pre-pregnancy BMI according to a systematic review by O'Brien et al. [21]. The drastic effects on cardiac, vascular, and endothelial function depend on the duration of obesity [22]. A population-based retrospective cohort study was conducted by Bicocca and colleagues to assess BMI at delivery and early and late-onset hypertensive disorders in pregnancy. They deduced that hypertensive disorders in pregnancy were significantly increased with an increase in BMI [23]. The risk of developing hypertension with obesity was also concluded from our study. Obese women are more likely to end labor induction with cesarean section, concluded Ellis et al., after a meta-analysis of eight studies. They also opined that women with higher BMI had a longer duration of labor induction involving larger and more frequent doses of both cervical ripening agents and oxytocin [24]. Surprisingly, in our cohort of obese patients, there was no increase in cesarean sections. This could be due to active management of labor in these patients as they will be labeled as high risk at the start of labor with one-to-one care. There were more primigravida patients in the normal weight group but even after adjusting for this factor, there was no increased risk of cesarean section in obese patients.

Postpartum hemorrhage was not increased in obesity according to studies by Butwick et al. and Siddiqui et al. [25,26]. We too made the same conclusion in our study. Postpartum complications including postpartum sepsis and wound infection were observed in our study. Maternal obesity had a risk of sepsis of 3.6/10,000 compared to 2.0/10,000 in women with a normal weight according to a retrospective observational cohort study in Sweden conducted in 1997-2012 by Axelsson and Blomberg [27]. An Australian study found overweight and obese women had a longer stay (>2 days) in the hospital in the antenatal period (21.6% of patients) [28]. Intervention does not necessarily influence the risks associated with obesity in pregnancy. Preventive measures to normalize body weight before embarking on pregnancy are therefore important [29]. Bariatric surgery causes physiological and anatomic changes associated with reproductive implications such as improved fertility and fecundity. Pregnancy outcomes are better than those who are not treated for obesity [30].

Key findings

Obesity was seen to be associated with older maternal age group women and with higher parity. Obesity increased pregnancy complications, which include hypertension, antepartum hemorrhage, and longer postpartum stay in the hospital.

Why the study was conducted

Maternal obesity highly influences maternal and fetal outcomes, which in turn affects future pregnancies of the women and has long-term effects on the mother and baby. Analyzing the effect of obesity is important for the preconception counseling of the population in order to organize their pre-pregnancy reduction of weight to optimize their obstetric outcome.

Strength of the study

Our study included a reasonable sample size based on our general population. Many factors were included such as antenatal assessment of oral glucose tolerance test (OGTT) in all pregnant women between 24-28 weeks of gestation or at the booking visit, blood pressure checkup, urine analysis at every antenatal visit, growth scan, and biometry assessment. Detailed analysis of the antepartum, intrapartum, and postpartum period helped us to identify the complications. Logistic regression was done to eliminate any confounding factors.

Limitations of the study

The study population can be too small to address some of the analyzed factors and the results need to be read with caution. Early pregnancy complications were not studied in this analysis. Although it is a completely different category of complications, it would be very relevant when the findings are used in the preconception counseling process. One of the limitations of any study addressing BMI is to analyze how much effort was put to try to change the BMI; for example, pre-conception counseling and dietician involvement during pregnancy.

## Conclusions

Obesity is a major health concern for pregnant women. The results of our study add to the literature on the consequences of obese mothers during antepartum, intrapartum, and postpartum periods. In our study hypertension, antepartum hemorrhage, and postnatal complications such as wound infection, sepsis, and prolonged hospital stay were found to be higher in the obese population. Public health practice should establish effective strategies to prevent obesity prior to pregnancy and focus on interventions to reduce obesity in women of reproductive age. Obstetricians need to oversee the pre-pregnancy counseling of obese women so that they gain normal BMI before embarking on pregnancy and relevant healthcare education on obstetric risks to the obese mothers at the antenatal booking visit. Weight reduction could be successfully achieved by pre-pregnancy surgical interventions, lifestyle modifications, and balanced diets during pregnancy. This level of awareness is necessary to reduce the adverse effects of obesity, besides the economic burden, and bring off improved obstetric outcomes. 

## References

[1] Kapoor E, Faubion SS, Kling JM (2019). Obesity update in women. J Womens Health (Larchmt).

[2] Okati-Aliabad H, Ansari-Moghaddam A, Kargar S, Jabbari N (2022). Prevalence of obesity and overweight among adults in the Middle East countries from 2000 to 2020: a systematic review and meta-analysis. J Obes.

[3] (2021). World Data Atlas: Bahrain - Female obesity prevalence as a share of female ages 18+. https://knoema.com/atlas/Bahrain/Female-obesity-prevalence.

[4] (2015). Royal College of Obstetricians and Gynaecologists. The role of bariatric surgery in improving reproductive health. RCOG Scientific Impact Paper on the role of bariatric surgery for improving reproductive health.

[5] Bautista-Castaño I, Henriquez-Sanchez P, Alemán-Perez N, Garcia-Salvador JJ, Gonzalez-Quesada A, García-Hernández JA, Serra-Majem L (2013). Correction: maternal obesity in early pregnancy and risk of adverse outcomes. PLoS One.

[6] Fitzsimons KJ, Modder J, Greer IA (2009). Obesity in pregnancy: risks and management. Obstet Med.

[7] Leddy MA, Power ML, Schulkin J (2008). The impact of maternal obesity on maternal and fetal health. Rev Obstet Gynecol.

[8] Mandal D, Manda S, Rakshi A, Dey RP, Biswas SC, Banerjee A (2011). Maternal obesity and pregnancy outcome: a prospective analysis. J Assoc Physicians India.

[9] Satpathy HK, Fleming A, Frey D, Barsoom M, Satpathy C, Khandalavala J (2008). Maternal obesity and pregnancy. Postgrad Med.

[10] Brite J, Laughon SK, Troendle J, Mills J (2014). Maternal overweight and obesity and risk of congenital heart defects in offspring. Int J Obes (Lond).

[11] Aune D, Saugstad OD, Henriksen T, Tonstad S (2014). Maternal body mass index and the risk of fetal death, stillbirth, and infant death: a systematic review and meta-analysis. JAMA.

[12] Denison FC, Aedla NR, Keag O, Hor K, Reynolds RM, Milne A, Diamond A (2019). Care of women with obesity in pregnancy: green-top guideline no. 72. BJOG.

[13] Bellver J, Rossal LP, Bosch E (2003). Obesity and the risk of spontaneous abortion after oocyte donation. Fertil Steril.

[14] Wang JX, Davies MJ, Norman RJ (2001). Polycystic ovarian syndrome and the risk of spontaneous abortion following assisted reproductive technology treatment. Hum Reprod.

[15] Fedorcsák P, Storeng R, Dale PO, Tanbo T, Abyholm T (2000). Obesity is a risk factor for early pregnancy loss after IVF or ICSI. Acta Obstet Gynecol Scand.

[16] Clark AM, Thornley B, Tomlinson L, Galletley C, Norman RJ (1998). Weight loss in obese infertile women results in improvement in reproductive outcome for all forms of fertility treatment. Hum Reprod.

[17] Reddy UM, Branum AM, Klebanoff MA (2005). Relationship of maternal body mass index and height to twinning. Obstet Gynecol.

[18] Sebire NJ, Jolly M, Harris JP (2001). Maternal obesity and pregnancy outcome: a study of 287,213 pregnancies in London. Int J Obes Relat Metab Disord.

[19] Weiss JL, Malone FD, Emig D (2004). Obesity, obstetric complications and cesarean delivery rate--a population-based screening study. Am J Obstet Gynecol.

[20] Marchi J, Berg M, Dencker A, Olander EK, Begley C (2015). Risks associated with obesity in pregnancy, for the mother and baby: a systematic review of reviews. Obes Rev.

[21] O’Brien TE, Ray JG, Chan WS (2003). Maternal body mass index and the risk of preeclampsia: a systematic overview. Epidemiology.

[22] Vasan RS (2003). Cardiac function and obesity. Heart.

[23] Bicocca MJ, Mendez-Figueroa H, Chauhan SP, Sibai BM (2020). Maternal obesity and the risk of early-onset and late-onset hypertensive disorders of pregnancy. Obstet Gynecol.

[24] Ellis JA, Brown CM, Barger B, Carlson NS (2019). Influence of maternal obesity on labor induction: a systematic review and meta-analysis. J Midwifery Womens Health.

[25] Butwick AJ, Abreo A, Bateman BT, Lee HC, El-Sayed YY, Stephansson O, Flood P (2018). Effect of maternal body mass index on postpartum hemorrhage. Anesthesiology.

[26] Siddiqui A, Azria E, Howell EA (http://2019). Associations between maternal obesity and severe maternal morbidity: findings from the French EPIMOMS population‐based study. Paediatric and Perinatal Epidemiology.

[27] Axelsson D, Blomberg M (2014). Prevalence of postpartum infections: a population-based observational study. Acta Obstet Gynecol Scand.

[28] Yang Z, Phung H, Freebairn L, Sexton R, Raulli A, Kelly P (2019). Contribution of maternal overweight and obesity to the occurrence of adverse pregnancy outcomes. Aust N Z J Obstet Gynaecol.

[29] Stubert J, Reister F, Hartmann S, Janni W (2018). The risks associated with obesity in pregnancy. Dtsch Arztebl Int.

[30] Wax JR (2009). Risks and management of obesity in pregnancy: current controversies. Curr Opin Obstet Gynecol.

